# Supplementary material for: Are carbon emissions trading and green financial instruments synergistic? -Comprehensive quantitative research based on content analysis
Source: PLoS One. 2024 Mar 7;19(3):e0298601. doi: 10.1371/journal.pone.0298601 (PMC10919601; doi:10.1371/journal.pone.0298601)
Supplement: S1 Data — (ZIP) [file pone.0298601.s001.zip › Data/地级市Z_F工作报告.zip!/╕╩╦α/└╝╓▌╩╨/└┤╫╘╣1⁄2╓┌║┼í╛┬φ┐╦╩2╛▌═°í┐_2009.docx]

各位代表：

　　我代表市人民政府向大会作工作报告，请予审议，并请各位政协委员和其他列席人员提出意见和建议。

　　一、2008年政府工作回顾

　　2008年是实施“十一五”规划的第三年，也是各种困难和矛盾比较多的一年。面对国内外宏观经济环境变化、低温雨雪灾害、“5·12”特大地震等带来的不利影响，我们在省委、省政府和市委的领导下，认真学习实践科学发展观，坚持好字优先、能快则快、增效增速、和谐发展，努力克服困难，狠抓工作落实，完成了市人大十四届三次会议确定的各项目标任务。

　　(一)经济保持平稳较快增长。在经历了“十五”以来主要经济指标低于和部分达到全省平均水平，2007年实现高于全省平均水平后，去年全市实现生产总值846.28亿元，比上年增长11.51%，增幅分别高于全国、全省2.51和1.41个百分点。完成全社会固定资产投资431.98亿元，增长20．46%。实现社会消费品零售总额395．04亿元，增长17．03%。完成大口径财政收入152.44亿元，增长19.05%；一般预算收入50．86亿元，可比口径增长25．44%；一般预算支出99．56亿元，增长19.52。城市居民人均可支配收入和农民人均纯收入分别增长13．68%和12．89%，达到11677元和3503元。居民消费价格总水平涨幅为7.2%，与全国、全省基本持平。全市金融机构各项存款余额、贷款余额和居民储蓄存款余额分别增长21.59%、17.11%和29.62%。节能减排成效明显，单位生产总值能耗下降6%以上，二氧化硫和化学需氧量排放总量分别削减13000吨和7000吨，超额完成了省上下达的指标。

　　(二)三次产业协调发展。一产、二产、三产结构比例为3.32：47.06：49.62。第一产业实现增加值28.1亿元，增长5.71%。粮食总产量达到38.75万吨，是五十年来第三个丰收年；蔬菜产量达到172.3万吨，其中外销80万吨。新增设施农业面积7500亩，双垄全膜覆盖种植面积达到31万亩。第二产业实现增加值398.25亿元，增长11.43%。工业增加值318.93亿元，增长13.16%，其中规模以上工业增加值296.59亿元，增长13.5%。工业占生产总值的比重达到37.69%，比上年提高1.13个百分点。装备制造业加快振兴，实现增加值43.13亿元，增长21.5%。实施了120项工业技术改造项目，建成39家省级以上企业技术中心，企业创新能力逐步提升。第三产业实现增加值419.93亿元，增长11.96%。启动了城区大型批发市场整合外迁工作，农副产品物流中心、粮油物流中心建设前期工作基本到位。大润发综合超市、众友医药物流中心建成运营，沃尔玛、王府井等大型项目正式签约。旅游业有了新的发展，全年接待国内外游客526.6万人次，旅游收入达到31亿元。非公有制经济加快发展，实现增加值287.6亿元，占全市生产总值的比重超过三分之一。

　　(三)重大项目建设进展良好。全市实施了100项重大项目，完成投资150亿元。兰州石化10万吨丁苯装罩、蓝星公司20万吨甲醇、兰州客运中心、黄河柴家峡水电站等项目建成运营，兰铝自备电厂、长城制造研发生产基地等项目基本完工，中核兰州铀浓缩、兰州石化丁腈橡胶、城市电网改造、天然气输配管网、甘肃会展中心等项目抓紧建设。工业企业出城入园的政策体系逐步完善，50户企业出城入园计划启动实施，西北永新集团等4户企业完成搬迁改造，12户企业正在开展前期工作。大力推进开发区建设和发展，高新区加快“二次创业”步伐，技工贸总收入达到400亿元，增长60%。经济区产业集聚功能不断增强，引进了康师傅饮品、深国投商业中心、奇正集团总部、中国华能集团西北总部等重大项目，技工贸总收入达到402亿元，连续三年实现翻番。大力推进院地校企合作，重点支持了15项产学研合作项目。兰州生物医药产业发展规划启动实施，总投资35亿元的44个项目完成前期工作，为申报国家级生物医药产业基地奠定了基础。特别是认真落实国家扩大内需、加大投资的宏观调控政策，及时筛选了三批500多个项目，积极向省上和国家部委汇报衔接，部分项目得到国家相关部委的支持，争取到位资金近10亿元。

　　(四)改革开放进一步推进。在全面完成国有企业改革主体任务的基础上，继续推进多种形式的企业资产重组，签约了兰州肉联厂股权转让等13个资产重组项目，与中石油昆仑燃气公司、首钢集团、中材集团达成战略合作协议，引进资金12.2亿元，盘活存量资产10．6亿元。企业上市工作力度加大，建立了30户企业资源信息库，基本完成佛慈股份、兰州银行等企业上市准备工作。城建系统50户企业脱钩改制进展良好，17户国有企业和26户集体企业完成改制任务。建立健全国有资产监管体系，出台了国有及国有控股企业综合绩效评价、企业国有资本经营预算管理等政策法规，成立了国有企业监事会工作机构。大力推进国有资产运营，为吉利轿车、金刚轮胎、蓝天玻璃等项目融资15亿元，实施了以城市规划用地置换兰州银行不良贷款工作，与中国东方资产管理公司达成了债务重组合作意向。积极探索企业非经营性资产运营的新路子，启动了破产企业危旧房屋改造工作，盘活存量土地234.7亩，拆迁安置危房住户1571户。新一轮政府机构改革进入前期准备阶段，完成了全市规范县级公安机构设置和人员编制工作。设立市级项目投资评审中心和政府金融工作办公室，理顺了非税收入、新型农村合作医疗、安全生产监督和节能监察等管理体制。全市水管体制改革任务基本完成，集体林权制度改革前期工作全面展开。进一步加大对外开放步伐，先后到全国25个城市学习考察和招商引资，成功举办了黄河文化旅游经贸洽谈会，全年签约各类国内合同项目286项，引进国内资金到位112.7亿元，引进国外资金9461万美元，引资总额创历史新高。实现进出口贸易总额7.15亿美元，其中出口5.86亿美元，增长3.57%。

　　(五)城市规划建设和环境治理取得新成效。第四轮城市总体规划修编工作全面展开，完成了城区北拓、老城区优化和城市色彩及空间特色、黄河主题雕塑长廊、南河道、三滩地区等重要规划设计，移山造地项目得到国土资源部和建设部的支持，沙中片30平方公里移山造地项目正式启动。实施了94项城市建设重点项目，完成投资37.33亿元。雁滩南河道整治二期和城市北出口、西北出口改造工程基本完工，草场街立交桥建成通车。总投资45亿元的庙滩子地区整体改造项目完成总体规划设计和前期工作。总投资46亿元的南山路项目全线开工建设。七里河安宁污水处理厂正式投入运营，西固、雁滩、盐场3个污水处理厂加快建设。榆中和平、七里河西津坪2个城市建筑垃圾处理场建成使用，皋兰忠和、七里河西津坪、安宁焦家庄3个生活垃圾处理场完成前期工作。城中村改造稳步实施，建成和开工建设村民安置房103万平方米。加大城市环境综合整治力度，全面完成城市环卫体制改革，推行了城市管理层级绩效考核。城市夜景亮化取得新成效，完成了黄河风情线一、二期亮化工程。城区空气环境质量持续好转，全年优良天数268天，冬季空气质量达到历史最好水平。黄河兰州段水质全面稳定达标，出境断面首次达到二类水质。黄河两岸整治项目获得“中国人居环境范例奖”。特别是针对城市管理方面存在的突出问题，集中开展“百日会战”、“六大整治行动”和客运市场营运秩序整治，市容市貌明显改观。城市经营工作进一步推进，与国家开发银行等金融机构达成近400亿元的融资合作意向，城投公司申请发行15亿元企业债券已经国家发改委同意。土地资源市场化运作有了新突破，全年完成协议储备土地1万多亩，出让土地950多亩，实现收益25.37亿元。

　　(六)社会各项事业稳步发展。全市城镇新增就业5.1万人，城镇登记失业率为2.8%，低于控制性目标1.2个百分点。输转城乡剩余劳动力27.6万人次。城镇基本养老、失业、医疗、工伤、生育保险实现应保尽保。低收入群体和困难群众的生活得到保障，17.4万城乡居民享受低保政策，发放保障金1.8亿元。完善了廉租住房保障制度，将人均住房面积8平方米以下的低收入家庭全部纳入保障范围，投入近1.2亿元解决了6966户城市特困家庭的住房困难。认真落实义务教育均衡发展政策，全市投入城乡义务教育“两免一补"资金9762万元。筹措资金6亿元实施地震受损中小学恢复重建，去年开工建设171所，建成使用141所。医疗卫生服务体系不断完善，新型农村合作医疗参合率达到92%以上，社区卫生服务覆盖面达到85%以上。公共卫生服务进一步加强，出台了突发公共卫生事件应急预案，及时稳妥地处置手足口病、婴幼儿奶粉事件，有效控制了疫病蔓延。文化事业健康发展，文物和非物质文化遗产保护工作得到加强，举办了秦腔艺术节等大型活动，建成6个乡镇综合文化站和240个“农家书屋”。全民健身活动蓬勃开展，基层公共体育设施建设力度加大，新增全民健身路径20条。人口和计划生育工作稳步推进，继续保持了低生育水平。完善了地震预测、预警、预防工作体系，全社会防震减灾意识明显增强。民族宗教、广播电视、外事、地方志、档案、人防、气象、残疾人等各项事业都取得了新的进步。

　　(七)为民兴办的实事全面落实。解决了8.3万人的安全饮水问题，超计划目标2.3万人；城镇居民基本医疗保险工作全面实施，61.3万城镇居民参加了保险，参保率达到86%；建成乡村公路1040公里，超计划目标340公里120万平方米经济适用住房和1500套廉租住房开工建设；50座城市公厕基本建成；新建农村生态家园沼气池7184座，超计划目标1684座；100条小街巷整治任务全部完成70个标准化村卫生所建成使用；出台了城乡低收入居民取暖补贴办法，将“三无对象”、城乡低保对象、低收入家庭全部纳入保障范围，根据供热价格调整情况及时提高取暖费补贴标准，安排供暖补贴3890万元，解决了近8万户、20万人的取暖问题30个放心粮店全部建成投入运营；改造新建10个标准化社区菜市场和10个定点屠宰厂；新增和改造城市绿地320公顷，超计划目标20公顷。

　　(八)民主法制和精神文明建设不断加强。自觉接受人大依法监督和政协民主监督，办结人大代表议案建议201件、政协委员提案710件。“五五”普法深入推进，政府立法工作进一步加强，制定修改地方性法规和政府规章11部，出台行政执法责任制相关配套制度。积极推进政务、村务、厂务公开和城乡居民自治，基层民主建设全面加强，完成了新一轮村委会和社区换届工作。加强机关作风建设，深入开展民主评议行风和纠风专项整治活动，政风行风明显好转。高度重视信访工作，认真组织开展大接访活动，解决了一批长期遗留的信访突出问题，维护了群众合法权益。强化审计监察工作，突出重点行业和重点事项的审计监督，完成各类审计项目289项。去年是我市精神文明建设取得丰硕成果的一年。“5·12”特大地震发生后，全市上下发扬“万众一心、众志成城，不畏艰险、百折不挠，以人为本、尊重科学”的伟大抗震救灾精神，一方有难、八方支援，积极组织公安、交通、卫生等部门参与灾区救助和恢复重建，向地震灾区捐款1．06亿元，捐赠物资价值2000多万元，极大地增强了全市人民的凝聚力和向心力。北京奥运圣火传递事关兰州的城市形象，我们加强组织领导，完善工作预案，多方协调配合，确保万无一失，使奥运圣火在我市安全顺利传递，受到了中央和省上的好评。“五城联创”工作取得积极进展，荣获省级历史文化名城和卫生城市称号，被中央文明委评为“全国创建文明城市工作先进城市”。双拥模范城建设实现“六连冠”。社会治安防控和应急管理体系进一步完善，治安秩序和安全生产形势持续好转，全市社会和谐稳定。

　　各位代表，过去的一年，我市经济社会发展取得的成绩来之不易。这是省委、省政府和市委正确领导的结果，是市人大、市政协监督支持的结果，是全市各族人民团结奋斗、共同努力的结果，也是坚定不移地推进改革开放的结果。改革开放三十年来，全市人民在艰苦创业中探索，在开拓、创新中进取，创造了令人瞩目的辉煌业绩，谱写了绚丽多彩的时代篇章。兰州的综合经济实力显著增强，人民生活水平不断提高，城市建设日新月异，社会各项事业全面发展，城乡面貌发生了巨大变化。全市生产总值由1978年的21．8亿元增加到2008年的846．28亿元，增长38倍；人均生产总值由1067元增加到25628元，增长23倍；一般预算收入由4.33亿元增加到50.86亿元，增长11倍；城市居民人均可支配收入由363元增加到11677元，增长31倍；农民人均纯收入由90元增加到3503元，增长38倍。三十年的建设成就，令全市人民为之骄傲和自豪；三十年的发展变化，为加快全面建设小康社会进程奠定了坚实基础。在这里，我代表市人民政府，向各位代表、政协委员，向各民主党派、工商联、人民团体和无党派人士，向离退休老同志和社会各界人士，向驻兰解放军指战员、武警官兵和公安民警，向多年来关心支持兰州现代化建设事业的同志们、朋友们表示衷心的感谢!

　　在肯定成绩的同时，我们也清醒地看到，全市经济建设、社会发展和政府工作中还存在一些矛盾和问题。一是经济增速减缓。去年9月份以来，全市工业有17个行业增速下滑，近70户企业停产或半停产，工业增加值增速比上年回落4．48个百分点，生产总值增速下降近1个百分点。二是建设资金紧缺问题比较突出。由于财力有限，加之市级财政配套保障范围不断扩大，刚性支出逐步增多，财政支出压力越来越大，城市基础设施建设、国有企业改革、中小学危房改造等项目存在较大资金缺口，部分项目实施进度有所放缓。这也是长期制约我市经济建设的一个突出矛盾。三是农民增收难度加大。受农资价格上涨、农产品销售困难、外出务工人员回流返乡等因素的影响，农民持续增收面临较大压力。四是房地产市场调整波动较大。全市商品房销售面积下降33．62%，二手房成交面积下降18．11%，房地产营业税增幅下降20多个百分点。五是节能减排任务艰巨。短期内我市治理高污染行业成效明显，但由于传统的经济和产业结构尚未根本转变，现代服务业和高新技术产业发展不快，经济社会发展受资源环境的约束较大。六是就业形势严峻。城镇新增就业人员和大中专毕业生增加，城乡劳动力输转难度增大，使得就业再就业矛盾十分突出。七是政府工作还需进一步加强。特别是社会管理和公共服务职能比较薄弱，少数工作人员责任感不强，工作效率不高，形式主义和消极腐败现象仍然存在，投资发展环境需要不断改善。这些问题，我们将高度重视，采取积极有效的措施，认真加以解决。

　　二、2009年的政府工作

　　2009年是贯彻落实党的十七大、十七届三中全会精神的重要一年，也是我市实施“十一五”规划、全面建设小康社会的关键一年。做好今年的工作，既有不利因素，也面临新的机遇。一方面，受金融危机影响，全球经济出现衰退，国际贸易和投资活动明显萎缩，世界经济将经历较长时间的低迷和调整；国内经济增速明显放缓，企业经营困难加剧，财政增收难度加大，经济运行将面临比较困难的局面。另一方面，国家扩大内需，实行积极的财政政策和适度宽松的货币政策，启动实施振兴十大产业规划，东部地区向西部地区产业转移步伐加快，将有利于我们争取更多的国家投资和项目，破解建设发展的融资难题，加快重点产业发展，推进产业调整升级，提高对外开放水平。综合分析各方面因素，政府工作的总体要求是：认真贯彻党的十七大和十七属三中全会精神，深入学习实践科学发展观，全面落实国家宏观调控政策，按照“一中心三率先、五加快五加强”的思路，以保增长、保项目、保民生、保节能减排、保稳定为重点，着力扩大投资和消费需求，切实转变发展方式，不断完善体制机制，坚定信心、共克时艰、趋利避害、能快则快，努力保持经济社会平稳较快发展。

　　经济社会发展的主要预期目标是：生产总值增长10%以上，力争达到11%；单位生产总值能耗降低5%，完成二氧化硫、化学需氧量总量控制等各项减排任务；全社会固定资产投资增长17%以上，力争达到20%；社会消费品零售总额增长16%；财政收入增幅高于生产总值增长水平；城市居民人均可支配收入增长10%，农民人均纯收入增长12%以上；城镇新增就业5万人，城镇登记失业率控制在4.6%以内；居民消费价格指数为104；人口自然增长率控制在5.5‰以内。

　　根据上述总体要求和预期目标，重点做好十个方面的工作：

　　(一)进一步加大项目建设力度，充分发挥投资拉动经济增长作用。坚持把国家扩大内需政策与兰州实际相结合，切实加大项目工作力度，努力保持投资较快增长。一是抓好重大项目建设。集中实施100个发展前景好、投资规模大、带动作用强的重大项目，突出抓好南山路、黄河大桥、沙中工业园、绿色生态文化中心、工业企业出城入园、农村基础设施及社会事业等项目的规划建设。各县区分别确定区域性重点项目，加大实施力度，抓好重点保障。二是积极做好项目争取工作。围绕城乡电网改造、生态环境建设、保障性安居工程建设等领域，筛选一批符合国家政策导向的新项目，加强与省上和国家有关部委的汇报衔接，争取部分项目列入国家投资计划。三是加强项目前期工作。完善项目工作机制，加大前期经费投入，强化项目的谋划、征集、筛选和论证，切实提高储备项目的数量和质量。重点抓好城市轨道交通、南绕城高速、窑街煤电煤化工、低温核供热、中川城镇综合改造和秦王川灌区农业综合开发等项目的前期工作。四是着力提高项目管理服务水平。进一步完善领导挂靠包抓项目责任制，加强项目规划、环境评估、征地拆迁协调力度，着力研究解决项。目建设中存在的突出问题。统筹兰州铁路枢纽及枢纽区域的规划建设和服务工作，确保兰渝铁路、兰州铁路枢纽及集装箱中心站、兰新客运专线、宝兰铁路客运专线、包兰铁路兰州—惠农二线、成都—兰州铁路等重大项目按计划实施。积极配合做好兰州—武威—张掖城际铁路、兰州—天水城际铁路、兰州原油商业储备库、涩宁兰复线、兰州—天水天然气管线等重大项目前期工作。

　　(二)坚持实施工业强市战略，保持工业经济平稳运行。按照率先实现新型工业化的要求，加快工业布局调整，促进产业结构优化，全市工业增加值增长13%左右。一是推动工业技术改造和产业升级。重点实施100项工业项目，建成兰州石化年产550万吨常减压装置、国电2×300兆瓦热电联产等项目，加快建设蓝星公司“1318工程”、榆钢二期等项目，力争开工建设金浦集团年产6万吨丁基橡胶和20万吨轻烃芳构化、蓝星公司多晶硅等项目。二是围绕园区建设培育新的增长点。加强对园区基础设施、技术创新和重点项目的倾斜支持，整合发展高新区雁滩园区、彭家坪新区、九州开发区、三川口开发区、石化产业园和空港产业园，加快项目、资金和高新技术产业向园区集中，高新区生产总值达到120亿元，力争“十一五”末在全国高新区排名从51位提升到35位左右，进入中等行列。经济区继续抓好基础设施建设，引进和实施一批重大产业项目，生产总值达到56亿元。坚持推进工业企业出城入园，完成6户企业搬迁改造。三是加快非公有制经济发展。全面落实中央和省市各项支持政策，消除制约非公有制经济发展的障碍，推动非公有制企业技术改造和创新，新增3户年销售额上3亿元的工业企业，23户科技成长型企业，180户年销售额上千万元的中小企业，非公有制经济增加值增长19%，达到340亿元以上。四是加强工业运行调控服务。积极做好企业生产经营的跟踪服务，认真落实土地、税收、财政支持政策，协调帮助解决融资和煤电油运等方面的困难，努力保证企业正常生产和经营。

　　(三)统筹城乡协调发展，加快推进城乡一体化进程。制定实施城乡一体化规划，以促进农民持续增收为核心，加快形成以工促农、以城带乡、统筹发展的新格局。一是加强农业基础设施建设。积极争取国家水利工程改造项目，启动实施三电、西电等大型泵站设备更新和灌区节水改造。加强引大灌区配套设施建设，完善已建调蓄水塘设施条件，新建一批调蓄水塘。继续抓好农村交通设施、安全饮水工程和生态家园沼气池建设。加大扶贫开发力度，完成20个整村推进项目。二是大力推进农业产业化。加快秦王川、小康营、青土坡、西岔川、西津坪5个千亩设施农业示范基地建设，全市新增设施农业面积6000亩以上，无公害农产品生产基地达到170万亩。推动“兰州高原夏菜”、百合、玫瑰、西甜瓜等特色产业发展，调整优化种植结构和区域布局，蔬菜总产量达到180万吨，外销量超过80万吨。积极推广农业先进技术和农作物新品种，双垄全膜覆盖种植面积达到35万亩。大力提高农业生产组织化程度，重点扶持10家示范性农民专业合作组织和10户农业产业化龙头企业。三是壮大县域经济实力。研究制定对县区放权让利的政策措施，赋予县区发展更多的自主权和财力支持。加快小城镇建设和发、展，抓好榆中和平全国发展改革试点镇工作，实施七里河阿干镇、西固河口、红古窑街、永登苦水、榆中甘草、皋兰西岔6个市列小城镇建设项目，增强小城镇的聚集辐射功能。四是抓好新一轮新农村建设试点工作。重点扶持40个试点示范村，着力发展特色产业，完善村庄规划，改善人居环境，强化公共服务。五是加大农村改革力度。努力形成农村土地承包经营权流转市场机制，鼓励有条件的县乡实行土地适度规模化和集约化经营。稳步推进林权制度改革。落实“一册明、一折统”惠农资金支付方式改革，切实保证各项支农惠农政策落到实处。

　　(四)加快现代服务业发展，完善城乡消费市场体系。发挥省会中心城市的集聚辐射功能，优先发展现代服务业，加快发展民生服务业，大力发展生产性服务业，着力培育发展新型业态，多渠道促进消费增长。一是构建集约发展的现代物流体系。抓好东部物流基地和陆港物流园区建设，加快实施城区大型批发市场外迁工程，整合搬迁蔬菜瓜果、水产肉食、粮油副食等批发市场。二是大力发展便民利民服务业。完善以菜市场、综合超市、放心早餐、放心粮油、再生资源回收为主的民生服务体系，促进便民消费进社区。积极推进牛肉面连锁经营，建成“中国兰州牛肉拉面馆"，逐步做大做强特色餐饮业。抓好粮、油、肉等重要大宗商品的储备调节，保证市场供应和物价基本稳定。三是积极发展节会经济。配合省上办好第十五届“兰洽会”，主动承办全国性大型展览会、交易会、博览会，组织好国内大型节会参展活动，以节会带动项目建设、促进经济发展。四是发展和繁荣农村市场。继续推进“万村千乡”市场工程，加快农村便利超市、农资超市和配送中心建设，着力扩大农村消费，搞活市场流通，带动农业产业化和地方特色经济发展。做好“家电下乡”等扩大农村消费政策的落实工作。五是推动旅游业健康发展。实施旅游业发展规划，打造丝路古道、黄河风情等精品旅游线路，实施青城古镇、鲁土司衙门世行贷款旅游项目，力争旅游收入增长15%以上。

　　(五)加大城市建设管理力度，进一步促进城市科学发展。围绕建设西部区域性现代化中心城市，坚持高起点规划、高标准建设、高效益经营、高水平管理，不断提高城市综合服务能力和辐射带动功能。一是加强城乡规划工作。加快第四轮城市总体规划和土地利用总体规划修编工作，突出城市布局调整、空间拓展、功能完善、产业发展和特色塑造，确保规划编制科学合理。开展东扩规划完善、黄河风情线优化提升、城市出入口美化改造等重大专项规划，实现中心城区控制性详细规划全覆盖。推进彭家坪、大滩、马滩、青白石、榆中盆地等重点区域开发建设。二是加大城市基础设施建设力度。计划投资50亿元，实施93项重点项目。路桥设施方面，抓好南山路、雁滩南河道整治三期、空港产业基地道路等项目，开工建设亚行贷款城市交通项目，争取轨道交通、南绕城高速前期工作有突破性进展。民生和城市安全方面，抓好小街巷整治、住房建设、地质灾害区域综合治理等项目，加快推进庙滩子地区整体改造。节能减排方面，抓好西热东输、东城区供热管网扩建、污水处理厂及配套管网建设等项目。环境整治方面，抓好重点区域的综合治理和品位提升，完成西关什字广场和邓家花园整治任务，启动大砂沟综合治理工程。城市经营方面，抓好世纪大道、雁滩、韩家河等区域的土地整理和开发项目。加快城中村改造步伐，做好土地出让、规划设计、招商引资等工作，建成村民安置房100万平方米。积极支持远郊县区城市建设，改善县城基础设施条件和服务功能。三是大力推进城市经营。统一整合旧城区土地资源，加大新城区和南山路等区域的土地储备力度，加快实施沙中片移山造地项目，收购储备土地1万亩以上。做好城市出入口、市区主干道、人行天桥、地下通道等户外广告经营权的拍卖转让和管理工作，实施金城关、白塔山、碑林一体化市场运营，加快七里河安宁污水处理厂TOT运作进度。四是切实加强城市管理。完善城关区数字化城市管理系统，加快近郊四区数字化管理网络建设。加大城市管理绩效考核力度，落实分级负责、门前三包等长效管理制度。做好城市垃圾处理费征收工作。健全环卫作业市场化运作办法，积极推行城市道路清扫对外招标管理。大力实施城市亮化、绿化、净化、美化工程，努力塑造山水城市新形象。

　　(六)强化节能减排和生态保护，着力改善城乡人居环境。坚持把节能减排作为转变发展方式的突破口来抓，建立健全政府主导、企业主体、社会参与的体制机制，加快建设资源节约型环境友好型城市。一是推动节能减排重点工程建设。实施兰州石化、兰铝、连铝等企业的51个资源综合利用和循环经济项目，完成方大炭素、兰州石化化肥厂等重点企业节能减排工程，抓好年耗能5000吨标准煤以上企业用能管理。严把项目建设能评环评准入关，把节能评估审查作为固定资产投资项目的强制性准入门槛，坚决淘汰落后产能，严格控制高耗能高污染行业增长。二是加强大气污染和黄河兰州段水污染治理。继续实施清洁能源“123计划”，完成城区1000家餐饮企业综合整治，空气质量优良天数达到80%左右。加快实施城市污水处理厂扩容及配套管网工程，强化重点污染企业和污染源监管，力争实现城市污水全收集、全处理。三是大力改善城乡地质环境。加强地质灾害防治，推进地质灾害监测预警示范项目建设，争取皋兰山等治理项目列入国家计划。四是加快城乡生态环境建设。做好南北两山林木管护和综合开发，推进黄河风情线景观文化长廊、500米半径小游园等项目建设，争取黄河中上游生态修
